# Infection resisters: targets of new research for uncovering natural protective immunity against *Mycobacterium tuberculosis*

**DOI:** 10.12688/f1000research.19805.1

**Published:** 2019-09-27

**Authors:** Vaishnavi Kaipilyawar, Padmini Salgame

**Affiliations:** 1 Center for Emerging Pathogens, Rutgers-New Jersey Medical School, International Center for Public Health, 225 Warren St, Newark, NJ, 07103, USA

**Keywords:** Innate immunity, M. tuberculosis, Infection resistors, Natural immunity, Tuberculosis, macrophages

## Abstract

“Infection resisters” are broadly defined as individuals who despite significant exposure to *Mycobacterium tuberculosis* remain persistently unreactive to conventional detection assays, suggesting that they remain uninfected or rapidly clear their infection early on following exposure. In this review, we highlight recent studies that point to underlying host immune mechanisms that could mediate this natural resistance. We also illustrate some additional avenues that are likely to be differently modulated in resisters and possess the potential to be targeted, ranging from early mycobacterial sensing leading up to subsequent killing. Emerging research in this area can be harnessed to provide valuable insights into the development of novel therapeutic and vaccine strategies against *M. tuberculosis*.

## Introduction

Tuberculosis (TB) is now the leading cause of death from a single infectious agent, *Mycobacterium tuberculosis* ^1^. An estimated 1.3 million TB-related deaths were reported globally among HIV-negative individuals in 2017 ^1, 2^. Nearly 23% of the world’s population is estimated to have a latent TB infection (LTBI), and 5 to 10% are at risk for progressing to active TB disease over the course of their lifetime ^3– 5^, consequently propagating the cycle of transmission. The lack of an effective vaccine, poor diagnostics and treatment management, along with the emergence of drug-resistant forms of *M. tuberculosis* have further sustained the global TB epidemic ^6^. Moreover, poorly characterized immune mechanisms of anti-mycobacterial host defense continue to challenge the development of robust host-directed therapeutic strategies.

The common tests for TB screening include the tuberculin skin test (TST) and the blood test interferon gamma release assay (IGRA), which test for prior exposure to mycobacterial protein antigens. In high-burden TB settings, following frequent and heavy *M. tuberculosis* exposure, the majority of infected individuals remain asymptomatic and TST/IGRA-positive and only around 10% progress to active TB disease ^4, 5^. Emerging evidence from several TB-endemic cohort studies involving active TB patients and their household contacts has identified another group of exposed individuals, referred to here as “resister” individuals, who never acquire infection or clear the pathogen and remain asymptomatic and persistently negative for TST and IGRA reactivity ^4, 7^. The prevalence of these individuals and their defining characteristics are described next.

### Evidence for the “resister” phenotype

Frequently exposed, persistently TST-negative “resister” individuals were identified as early as 1937 among student nurses at the Boston City Hospital (Boston, MA, USA) who were annually screened for TB over a five-year study period ^8^. Around the same time period, they were also reported to be present among students who frequently dealt with TB patients in other hospitals such as the Wisconsin General Hospital ^9^ and the University of Minnesota ^10^. In 1966, enlisted personnel onboard the naval ship *USS Richard E. Byrd* shared the same work areas with an index case of pulmonary TB. About 10% were negative for the purified protein derivative (PPD) skin test despite being heavily and frequently exposed in a closed environment, while most personnel showed a positive PPD accompanied by LTBI or the development of active TB ^11^. Identification of resisters among close contacts of pulmonary TB patients in India, Pakistan, and Bahia has also been reported ^4^. Together, these studies clearly indicate that there is evidence for the existence of resister individuals among the exposed. However, the frequency of occurrence of resisters has differed across such population studies since the defining characteristics of the resister phenotype have been largely inconsistent.

### Identification and classification of resisters

Several caveats exist when it comes to correctly identifying and classifying the resister phenotype. The very definition of this phenotype makes the assumption of having repeated exposure to infectious doses of *M. tuberculosis* and is based on the reactivity to the TST and IGRA tests ^4, 5^. However, a localized protective immune response within the lung mucosa may not be systemically detected by these tests. Furthermore, these tests do not reflect or quantify the level of pathogen exposure or the status of infection and it has been argued that resisters simply have not had sufficient exposure to an infectious dose. Nevertheless, cohorts where contacts had a comparable intensity of exposure still identify this phenotype, suggesting that criteria for study enrollment need to be more stringent to avoid misclassification ^4, 7^. The presence and prevalence of “true” resisters thus can be reasonably estimated in future cohort investigations by employing a longitudinal follow-up period that is supported by repeat testing to minimize false negatives and taking into account the duration and intensity of exposure to the index case ^4^. Additionally, the influence of age, gender, co-morbidities, and environmental factors needs to be considered when evaluating the resister phenotype.

### Host innate immune responses against *Mycobacterium tuberculosis*

Conventionally, experimental models have shown that, following exposure and infection of the airways and lung parenchyma, the early host immune response to *M. tuberculosis* involves an influx of phagocytic cells such as mononuclear cells and neutrophils ^5, 12^. Alveolar macrophages (AMs) are the primary cellular niche for intracellular *M. tuberculosis* survival and chemokines secreted by infected AMs initiate immune cell recruitment to the lungs for bacterial clearance during early infection ^13, 14^. Induction of anti-mycobacterial mechanisms, such as production of pro-inflammatory cytokines, reactive oxygen and nitrogen intermediates, anti-microbial peptides, phagosome acidification, and autophagy induction then facilitate bacterial killing ^5, 13, 15^. Infected macrophages are also known to undergo apoptosis to restrict *M. tuberculosis* growth and enhance antigen presentation by dendritic cells, inducing an early *M. tuberculosis*–specific interferon gamma (IFN-γ)-mediated type 1-helper (Th1) T-cell response ^16– 18^. Recent studies in mice and non-human primate models suggest that innate-like T cells ^19– 21^ and humoral immunity ^22– 24^ also play a protective role in the control of intracellular pathogens.

Since resisters display persistent TST/IGRA negativity, it can be postulated that robust innate immune responses enable early mycobacterial clearance in these individuals and circumvent the need for the conventional IFN-γ–dependent T cell–mediated adaptive immunity observed in LTBI. Additionally, the screening tests have been widely regarded as incomplete measures of an anti-mycobacterial response because they do not detect unconventional IFN-γ–independent cytokine responses, antibody-mediated responses, or immune responses directed against non-protein mycobacterial antigens ^4, 5, 25^. Despite repeated exposure to *M. tuberculosis*, mycobacterial resistance could be established as a result of such largely overlooked immune responses and remain uncharacterized. Although genetic susceptibility to *M. tuberculosis* assessed by genome-wide analyses has associated factors such as polymorphisms and mutations in influencing TST reactivity or Toll-like receptor (TLR) pathways, the broad functional effects of such elements are unknown in the context of infection ^4, 26, 27^. Emerging evidence also strongly indicates that genetic variation among *M. tuberculosis* strains significantly impacts the host immune response, enabling immune evasion strategies that promote mycobacterial replication, dissemination, and subsequent transmission ^28, 29^. Thus, understanding the mechanistic basis of the early events leading up to the establishment of resistance will allow the identification of correlates of protection which can be harnessed to develop novel host-directed therapeutic strategies.

This review will further summarize recent advances in the context of resistance to *M. tuberculosis* infection and also highlight the potential involvement of alternate pathways that might contribute to rapid mycobacterial clearance observed in frequently exposed, persistently TST- and IGRA-negative individuals. We will also discuss the implications of such findings and how they pertain to the development of robust host-directed therapeutic strategies.

## Recent findings from tuberculosis-endemic cohort studies

Owing to the severity of infection observed in the absence of IFN-γ ^30^, it is often thought to be the chief mechanism by which the host controls *M. tuberculosis* infection. However, as discussed earlier, there is increasing evidence that IFN-γ is not a robust correlate of protection ^31^ and that alternate mechanisms of inducing protective immunity exist in resisters. In this section, we will further highlight recent reports from various cohorts that have sought to identify and elucidate some of these protective immune mechanisms in resisters, providing significant insights into factors that potentially mediate mycobacterial resistance.

### Transcriptional response of monocytes (Kampala, Uganda)

A longitudinal cohort established in Uganda identified 872 index cases and 2585 household contacts, of whom 255 (9.9%) were persistently TST-negative on repeated testing over two years of follow-up ^32^. Genome-wide transcriptional profiling was conducted by using peripheral blood monocytes from resister and LTBI individuals who were infected *ex vivo* with *M. tuberculosis* H37Rv. With a systems biology approach, gene sets associated with the histone deacetylase (HDAC) function were found to be the top differentially activated genes among these groups. Treatment of U397 monocytes with small-molecule inhibitors of HDAC, depsipeptide, and sodium butyrate suppressed the secretion of pro-inflammatory cytokines interleukin-1 beta (IL-1β), IL-6, and tumor necrosis factor (TNF) against *M. tuberculosis* infection ^32^. These data further indicate that HDAC function might be a vital player modulating the early innate immune response in resisters, and whether epigenetic modifications in response to infection lead to distinct transcriptional modifications should be further explored.

### Functional response of innate T-cell subsets (Port-au-Prince, Haiti)

Between 2015 and 2017, a cross-sectional study conducted in Haiti enrolled 92 household contacts of pulmonary TB patients and 591 community controls which were sampled to obtain an age-, sex-, and IGRA-matched subset of 31 TB household contacts and 45 unexposed community controls ^33^. Exposed but uninfected IGRA-negative resisters (12 out of 31; 39%) showed no IGRA-positive conversion after a 6-month follow-up. The abundance and functional profiles of innate T cells including Mucosal-associated invariant T (MAIT) cells that recognize riboflavin pathway metabolites; γδ T-cell receptor (TCR) T cells that recognize phosphoantigens; and invariant natural killer T (iNKT) cells that recognize glycolipids via their CD1 molecules, were compared among contacts and their community controls. There were no differences in the abundance of innate T cells in contacts compared with controls; however, resisters were shown to have robust CD8 ^+^ MAIT cell IL-2Rα chain (CD25) expression and granzyme B production upon CD3-TCR stimulation, which also was associated with a depressed CD69 and IFN-γ response when compared with LTBI controls ^33^. Additionally, *M. tuberculosis* exposure was found to be associated with differences in gut microbial composition across households, correlating with MAIT cell abundance and function, also suggesting a role for the intestinal microbiome in modulating innate T-cell subset responses ^33^.

### Protective antibody responses in health-care workers (Beijing, China)

In a study conducted at the Beijing Chest Hospital, IgG antibodies were isolated from 48 health-care workers (also comprising exposed yet IGRA-negative workers) who worked at the hospital for at least three years and who were compared with 12 patients with active TB ^24^. Health-care workers were categorized as having LTBI or “highly exposed but uninfected” (HEBUI) on the basis of enzyme-linked immune absorbent spot (ELISpot) testing. Importantly, antibodies from seven health-care workers passively conferred moderate protection against a low-dose *M. tuberculosis* mouse aerosol infection model, three of whom were IGRA-negative while the rest had evidence of LTBI. With an *in vitro* whole blood assay, three donors (including one HEBUI donor) were shown to neutralize *M. tuberculosis* and offer 2- to 3.3-fold protective antibody responses. These antibody responses were targeted against mycobacterial surface antigens and were shown to be dependent on immune complexes and CD4 ^+^ T cells for their functional efficacy ^24^. Their findings further suggest that since CD4 ^+^ T cells were required for efficacy, they either are not specific for conserved antigens ESAT-6 or CFP-10 or do not secrete IFN-γ in response to antigen.

### Innate cells and cytokines associated with early clearance (Bandung, Indonesia)

Among 1347 exposed TB case contacts enrolled in a cohort study in Bandung, Indonesia, 317 were identified as “early clearers” who remained persistently IGRA-negative and 116 were identified as “converters” at 2-week and 14-week post-enrollment by repeat IGRA testing ^34^. Flow cytometric analysis of immune cell populations demonstrated a profile associated with the resolving innate cellular response from 2 to 14 weeks in persistently IGRA-negative contacts but not converters. There were no differences in cytokine responses to mycobacterial stimuli among these two groups of individuals, but compared with converters, persistently IGRA-negative contacts produced more pro-inflammatory cytokines when stimulated with other pathogens such as *Escherichia coli* and *Streptococcus pneumoniae*. The authors further hypothesize that early clearance is mediated by epigenetic and cellular metabolic changes that may be a consequence of trained immunity ^34^. The authors also emphasize that early clearers in this cohort had a lower exposure to *M. tuberculosis* when compared with converters and would have led to misidentification, highlighting the need to have stringent criteria to identify and define resisters.

### Interferon gamma–independent responses (Kampala, Uganda)

Recently, IFN-γ–independent response was the subject of investigation in a re-tracing study of the Ugandan cohort described previously. Of the 441 (63.8% of the original 2014–2017 cohort) individuals who consented to the re-tracing study, 82 resisters and 195 LTBI individuals were definitively identified on the basis of concordant reactivity to repeat TST and IGRA testing ^23^. It was demonstrated that “resisters” possess IgM and class-switched IgG antibodies that were skewed toward IgG1 as observed in subclass ratios when compared with LTBI controls, further indicating that resisters possess affinity-matured antibodies in response to prolonged exposure to *M. tuberculosis*. PPD-specific, Fc-glycan profiles were different across the groups, and resisters demonstrated elevated levels of singly galactosylated (G1), highly fucosylated, bisected, and decreased sialylation. Importantly, these resisters also displayed detectable ESAT6/CFP10-specific CD4 ^+^ T-cell responses characterized by the absence of IFN-γ but the presence of TNF ^+^IL-2 ^+^CD40L/CD154 ^+^, IL-2 ^+^CD40L/CD154 ^+^, CD40L/CD154 alone or CD107a alone T-cell subsets, indicating the involvement of an unconventional IFN-γ–independent adaptive immune response ^23^. The authors propose that a distinct Th pathway (T-follicular, Th2, Th17, Th1) is likely induced in response to differential exposure that influences major histocompatibility complex–mediated antigen processing and presentation in response to *M. tuberculosis* infection.

Collectively, these reports strongly suggest that resisters possess distinct immunological mechanisms that allow them to remain uninfected. However, whether a single dominant mechanism or multiple complementary mechanisms give rise to the protective response in resisters is still unclear. In the next section, we discuss additional immune mechanisms that could also mediate early and rapid clearance of *M. tuberculosis*.

## Additional effector mechanisms modulating the establishment of mycobacterial resistance

### Responses mediated by the lung mucosa

The respiratory epithelium is essentially the first line of defense against pathogens since it serves as a physical and functional barrier that is actively involved in pathogen clearance. The innate pulmonary host defenses are supplemented by the anatomical structures of the conducting and peripheral airways which are lined by diverse populations of epithelial cells and submucosal glands ^35^. Additionally, epithelial cells recognize pathogen-associated molecular patterns and danger-associated molecular patterns via their pattern-recognition receptors (PRRs) that include plasma membrane TLRs and soluble cytosolic Nod-like receptors and initiate signaling to recruit immune cells ^36, 37^. Following inhalation, the most common route of *M. tuberculosis* transmission occurs following the deposition of bacilli in the alveolar sacs within the lung. The alveolar compartment is lined with pneumocytes such as squamous type I alveolar epithelial cells (AEC I) and cuboidal type II alveolar epithelial cells (AEC II) ^20^. While AEC I facilitate gas exchange and take part in sensing microbial products, AEC II also function as non-professional antigen-presenting cells and secrete a broad variety of cytokines and chemokines that are involved in activation and differentiation of immune effector cells ^20, 38^. It is unclear whether *M. tuberculosis* can invade and replicate within AEC II or other respiratory epithelial cells during natural infection; however, A549 lung epithelial cells infected with *M. tuberculosis* bacilli have been observed to be rapidly killed *in vitro* ^39, 40^. Such initial mycobacterial interactions with the lung epithelium could shape the course of the ensuing innate immune effector response, thus establishing early mycobacterial resistance. Genetic susceptibility to *M. tuberculosis* has been associated with polymorphisms in pathogen-sensing TLR pathway mediators such as Toll-interacting protein (TOLLIP) and TST1 and TST2 loci that influence TST reactivity ^4, 26, 27^. Inhibition of *M. tuberculosis* uptake to prevent infection is an obvious target for host-directed therapy; however, the presence of extracellular bacilli would likely dampen the host’s ability to kill these bacilli. In addition to surface PRRs, cytosolic detection of microbial DNA and cyclic di-nucleotides occurs via cGAMP synthase (cGAS) binding of microbial DNA to produce 2'3'-cyclic GMP-AMP (cGAMP) and activates stimulator of interferon genes (STING), making both STING and cGAS key targets of investigation for potential therapeutic manipulation of responses from the lungs ^41^. It remains unclear whether inhibitors of surface PRRs and cytosolic sensors are sufficient to impair subsequent bacterial pathogenesis and warrant comprehensive examination.

Lung epithelial cells are known to secrete soluble surfactant proteins (SPs) complement proteins, lysozyme, and anti-microbial peptides (APs) such as cathelicidin peptide LL-37 and β-defensins into the alveolar space, further indicating that such secretory proteins possess the potential to aid in early pathogen clearance ^42, 43^. SP-A and SP-D polymorphisms are known to be associated with susceptibility to TB disease ^44^. In fact, in a case-controlled study of 364 patients with TB and 177 control subjects in Taiwan, the SP-D 92T homozygous genotype was found to be a risk factor for TB ^45^. *In vitro* assays further supplemented this finding by demonstrating that this variant had a lower binding ability to *Mycobacterium bovis* as well as a lower capacity to inhibit phagocytosis, resulting in less inhibition of intracellular growth of *M. bovis* ^45^. It remains to be elucidated whether such polymorphisms exist in resisters and the consequent functional effects on early clearance. Additionally, APs such as the β-defensins HBD-2 and HDB-4 are expressed in response to activation of epithelial TLRs and decreased levels of these APs were shown in airways of individuals with chronic obstructive pulmonary disease ^35, 46^. Although the protective roles of APs are well characterized in the context of inflammatory skin disorders ^47– 49^, there is emerging evidence that APs do play a dynamic role in *M. tuberculosis* control ^50– 52^. AP expression has been shown to be induced by the IL-17/IL-22 cytokine axis as well as IL-1 cluster of cytokines such as IL-36γ ^47, 53, 54^. These cytokines have also been shown to induce Th1 immune responses, which are critical for controlling intracellular bacterial pathogens ^55– 57^. In THP-1 alveolar macrophages, IL-36γ–induced APs restrict *M. tuberculosis* H37Rv growth ^58^. It is likely that pathogen sensing by the respiratory mucosa induces distinct functional pathways resulting in the production of APs that are able to successfully restrict *M. tuberculosis* growth early on following exposure, either via direct neutralization or via indirect pathways, and need to be mechanistically defined in resisters. In this regard, vitamin D (Vit D) has been the subject of several investigations in its ability to induce cathelicidin anti-microbial peptide (CAMP) via signaling through the Vit D receptor ^59^. Some studies have demonstrated that Vit D deficiency was associated with an increased risk of TB and that Vit D supplementation in fact improved *M. tuberculosis* growth restriction in patients when compared with controls ^60^. Class I HDAC inhibitors that modify chromatin and cell signaling are also known to induce cathelicin production and restrict *M. tuberculosis* growth by synergizing with Vit D ^4, 61^. A summary of findings from clinical trials evaluating Vit D therapy ^62^ details insights from other respiratory infections, providing inferences to strengthen future experimental designs and investigations for *M. tuberculosis*. Natural and synthetically engineered APs represent potential therapeutic agents that should be evaluated for their potential to neutralize or restrict *M. tuberculosis* growth ^63^.

Alveolar macrophages, MAIT cells, and tissue-resident memory T cells represent some of the immune cell populations that reside within the lung ^20^. These cell populations have been shown to be induced and maintained long-term following initial pathogen exposure and are able to rapidly induce *M. tuberculosis*–specific recall responses that provide immediate and effective protection at the portals of entry ^64– 66^. Thus, protection mediated by such lung-resident immune cells may not be reflected in the systemic immune response and might be the dominant protective immune mechanism that is employed by resisters. Experimental mouse models further complement this hypothesis and have shown that accelerating effector cell production and delivery to lung in primary *M. tuberculosis* infection improves the infection outcome ^65^. Recently, it was demonstrated that subcutaneous vaccination of rhesus macaques with cytomegalovirus vectors encoding *M. tuberculosis* antigen inserts was able to elicit and maintain differentiated, circulating, and tissue-resident *M. tuberculosis*–specific CD4 ^+^ and CD8 ^+^ memory T-cell responses ^67^. The overall extent of infection and disease was reduced by 68% compared with unvaccinated controls after intra-bronchial *M. tuberculosis* challenge ^67^. Although the development of strong experimental models that mimic the local lung environment in resisters is challenging, vaccine therapies that are able to induce a robust effector response within the lung should be the subject of future research.

### Macrophage-mediated *Mycobacterium tuberculosis* growth restriction

Following exposure and infection, macrophages are at the forefront of orchestrating the immune response against *M. tuberculosis*, facilitating immune cell recruitment from peripheral blood for early pathogen clearance or containment ^4, 5^. Furthermore, macrophages are widely regarded as the primary niche for *M. tuberculosis* growth and survival ^5^ and it is likely that macrophage-dependent pathways could clear initial *M. tuberculosis* infection in resisters before the development of an adaptive immune response. While apoptosis of infected macrophages represents an important innate host defense modality limiting the viability of intracellular *M. tuberculosis*, necrosis is often employed as a virulence strategy by *M. tuberculosis* to promote mycobacterial dissemination ^16, 68– 70^. Pro-inflammatory eicosanoids prostaglandin E (PGE) and anti-inflammatory lipoxin (LX) have been shown to modulate macrophage apoptosis and are critical to determining the outcome of *M. tuberculosis* infection in the host ^18, 71^. Interestingly, prostaglandin E synthase (Ptges) ^-/-^ mice and prostaglandin receptor EP2 ^-/-^ were shown to have increased susceptibility to *M. tuberculosis* infection ^72^, suggesting that PGE _2_ and the apoptotic death of macrophages might be critical in regulating *M. tuberculosis* growth *in vivo*. Another study showed that 5-lipooxygenase ( *Alox5 ^-/-^*) mice lacking the enzyme required for both pro-inflammatory leukotriene (LT) and LX biosynthesis exhibited significantly lower *M. tuberculosis* lung burdens when compared with wild-type mice ^73^. The zebrafish *Mycobacterium marinum* model identified that variations in LTA _4_H levels influence the balance between pro-inflammatory LTB _4_ and anti-inflammatory LXA _4_^74^. Collectively, these studies suggest that striking the proper balance between the levels of eicosanoids influences the modality of cell death and might be critical for the successful early control of *M. tuberculosis*. Extensive studies are necessary to determine whether the two forms of cell death are differentially regulated in resisters, subsequently affecting mycobacterial growth and pathogenesis *in vivo*.

In contrast to apoptosis and necrosis, autophagy is a fundamental method of maintaining homeostasis via the degradation of cytoplasmic contents in lysosomes and has recently been established as an innate immune defense pathway in mouse models of TB autophagy ^75– 77^. Protein aggregates or defective organelles are sequestered by double-membrane structures called isolation membranes or phagophores, which mature into autophagosomes that are capable of fusing with lysosomes ^76^. Mammalian target of rapamycin (mTOR) kinase negatively regulates autophagosome generation by targeting the initiation complex components such as the PI3KC3 (class III phosphoinositide 3-kinase complex 3), ULK1 (unc-51-like kinase 1) complex, and ATG complex. Inactivation of the mTOR kinase is likely to specifically impact autophagy ^78^. For this reason, mTOR inhibition therapy has recently garnered interest in inducing autophagy as a protective mechanism against *M. tuberculosis* ^79^. Conventional anti-microbial peptides such as cathelicidin and neo-anti-microbial peptides known as cryptides are generated as a result of autophagy that aid in intracellular *M. tuberculosis* killing ^75^. IL-1β induces autophagy in a MyD88-dependent fashion and promotes autophagosomal maturation into degradative autolysosomes ^80^, signifying a potential role for IL-1β in innate mechanisms of host defense, although the extent to which IL-1β regulates the autophagy pathway and induction remains to be studied.

Interestingly, IL-1 and type I IFNs represent two major counter-regulatory classes of inflammatory cytokines that control the outcome of infection ^18^. Induction of type I IFN is pathogenic in animal models of *M. tuberculosis*, and increased type I IFN activity correlates with active disease in humans ^81^. For example, mice lacking IFNAR survive longer than wild-type mice after *M. tuberculosis* infection ^82^. In infected mice and patients, reduced IL-1 responses or excessive type I IFN induction or both are linked to an eicosanoid imbalance associated with disease exacerbation ^18, 83– 85^. In two human cohorts, patients with a lower PGE _2_-to-LXA _4_ ratio had worse sputum grade ^86^. Moreover, mice lacking IL-1α and IL-1β expressed high type 1 IFN levels and treatment of these mice with a 5-LOX inhibitor, together with PGE _2_, led to decreased lung pathology and bacterial burden ^86^. Taken together, these observations suggest that type 1 IFN responses might be suppressed in resisters and might instead play a prominent role in establishing LTBI or active TB disease and need to be mechanistically defined in future studies.

### Responses mediated by innate-like lymphoid cells

Whereas conventional T cells exhibit a delayed onset upon primary infection and are clonally heterogeneous among human populations, innate-like T cells rapidly respond to pathogens and secrete cytokines without undergoing extensive clonal expansion to induce unique anti-mycobacterial immune defenses ^87– 89^. The Haitian cohort described previously demonstrated that MAIT cells, but not iNKT cells or γδ T cells, display evidence of prior activation ^33^. Recently, changes in peripheral levels of natural killer (NK) cells (that are known to induce non-major histocompatibility complex–restricted cytotoxicity) were shown to indicate disease progression and treatment responses ^90^. NK cell levels were found to inversely correlate with the inflammatory state of the lungs of TB patients across three longitudinal cohort studies ^90^. However, it is unclear how all of these cell types respond to *M. tuberculosis* infection and whether they selectively proliferate in response to the presence of the pathogen. Additionally, in the context of an *M. tuberculosis* infection, innate lymphoid cells (ILCs) are a lesser-studied population. ILCs are broadly classified into three main subsets: ILC1 (IFN-γ–producing), ILC2 (IL-4–, IL-5–, and IL-13–producing), and ILC3 (IL-17– or IL-22–producing or both). In the lung mucosa, ILC3s are an abundant lymphoid cell progenitor population that rapidly respond to microbial and cytokine signals ^19^. A significant reduction in all ILC populations was reported among 44 subjects with diagnosed active drug-susceptible and drug-resistant TB infections in comparison with healthy controls ^37, 91^. Recently, it was demonstrated that circulating subsets of ILCs (ILC1s and ILC3s) are depleted from the blood circulation in patients with pulmonary TB and their numbers are restored following treatment ^21^. ILCs were found to be present in lung tissues from participants with active TB disease and transcriptional profiling of ILCs isolated from these tissues further revealed genes associated with a coordinated response against *M. tuberculosis* infection. Furthermore, in a C57BL/6 mouse model, ILC3s accumulated in the lung, coinciding with the accumulation of alveolar macrophages, associating with lymphoid follicle-containing granulomas. Their accumulation was accompanied by the upregulation of CXCR5 on ILC3s and increased plasma levels of its ligand CXCL13. With *Il17 ^-/-^ Il22 ^-/-^* double-knockout mice, IL-17 and IL-22 were found to be critical inducers of CXCL13 and impaired ILC3 responses resulted in an increased bacterial burden ^21^. Although collectively these reports implicate a role for innate-like T cells in modulating the immune response, the roles and extent to which these cells contribute to early mycobacterial restriction in resisters remain to be delineated. Moreover, alternative mechanisms such as innate-like B1 cell mediated, T cell–independent responses ^92, 93^ are yet to be investigated. Such innate responses in resisters might also be directed predominantly against non-protein mycobacterial antigens ^94^, possibly circumventing the development of the classic CD4 ^+^ T cell–mediated IFN-γ response, providing a reasonable explanation for persistent TST/IGRA negativity observed in these individuals. These unconventional immune responses involving IFN-γ–independent cytokine responses are described next.

### Alternate cytokine-mediated responses

As discussed earlier, IFN-γ responses do not correlate with better protection against *M. tuberculosis* infections ^31^. For example, mice deficient in V(D)J recombination activating protein (RAG) that received bacillus Calmette–Guérin (BCG)­specific Th17 cells from immunized IFN-γ–deficient mice had a survival advantage when challenged with *M. tuberculosis*, levels of which were comparable to that seen with transfer of Th1 cells from IFN-γ–competent mice ^95^. Indeed, circulating levels of IL-17 have been shown to be lower in patients with active TB than in those with LTBI ^96^. In a study from Gambia, following whole blood stimulation with *M. tuberculosis* antigens, Th17, Vγ9Vδ2 ^+^, and CD161 ^++^Vα7.2 ^+^ MAIT cells were analyzed by flow cytometry ^97^. The majority of IL-17 was produced by CD26 ^+^CD4 ^+^ Th17 cells, followed by γδ T cells (6.4%) and MAIT cells (5.8%), and IGRA-negative subjects demonstrated significantly higher levels of IL-17A, IL-17F, IL-21, and IL-23 in antigen-stimulated supernatants. In another comparative study analyzing TST-positive and TST-negative subjects, TST-positive individuals showed a downregulation of IL-17, IL-23, and RORγt (a key transcription factor for Th17 cells) but no difference in Th1 and Th2 cytokines ^98^. In agreement with IFN-γ–independent immune responses observed in the Ugandan cohort ^23^, these reports further indicate that IFN-γ alone might not be sufficient for the protective immune response and that elevated levels of IFN-γ might instead be unfavorable for optimal protection. IFN-γ–independent immune responses that are in fact IL-17–mediated could also be orchestrating the establishment of resistance via IL-17–producing innate T cells described previously and need to be extensively investigated.

Other cytokines, such as the anti-inflammatory cytokine IL-10, are known to mitigate Th1 cell responses and minimize pro-inflammatory effects of TNF and IFN-γ. Cambodian pulmonary TB patients who remained anergic to PPD following treatment completion displayed tuberculin antigen-specific T-cell responses ^99^. These responses were characterized by the production of IL­10 rather than IFN-γ ^99^. Another study, in Ghana, found that individuals with the highest association with the IL-10 promoter haplotype had low circulating levels of IL­10 and were more likely to have TB or be TST­positive ^100^. In an *in vitro* study, bioactive *M. tuberculosis* cell wall fragments induced upon mycobacterial contact with human alveolar lining fluid were shown to prime human macrophages to better control *M. tuberculosis* infection through an increase of phagosome–lysosome fusion events in an IL-10–dependent manner ^101^. Experimental models that recapitulate the early cytokine response to *M. tuberculosis* must be developed to identify the roles of IL-10 and other lesser-studied cytokines involved in mycobacterial growth restriction in resisters, providing insights for the potential of cytokine manipulation as a preventive therapeutic strategy.

### Trained immunity and bacillus Calmette–Guérin revaccination

Although adaptive immunity is widely accepted to possess immunological memory, recent studies have demonstrated that innate immune cells are able to resist reinfection with the same or an unrelated pathogen via a phenomenon termed “trained immunity” that is driven by differential gene expression induced by epigenetic modifications ^102, 103^. Epidemiological studies have further shown that BCG vaccination induces non-specific protection that is effective through early childhood (reviewed in 104). Moreover, it has been postulated that exposure to *M. tuberculosis* itself is likely to induce trained immunity ^104^ and that, owing to the presence of environmental mycobacteria in TB-endemic settings, bolstered trained immunity in resisters could likely prevent mycobacterial infection. Upon BCG vaccination, peripheral blood mononuclear cells and isolated NK cells are known to produce increased levels of pro-inflammatory cytokines TNF and IL-1β in response to *M. tuberculosis* as well as other pathogens such as *Candida albicans* and *Staphylococcus aureus* ^104^. Importantly, when healthy individuals were vaccinated with BCG or a placebo and subsequently with the live attenuated yellow fever vaccine, a decreased peak yellow fever viremia was observed ^105^. This response correlated with increased production of IL-1β ^105^. In another study, enhanced mycobacterial growth restriction was observed upon BCG vaccination in a subset of “responder” individuals who demonstrated a differential DNA methylation pattern among genes belonging to immune pathways ^106^. Furthermore, it was shown that the shift of glucose metabolism toward glycolysis is a fundamental process in trained immunity, inducing key histone modifications and functional changes, emphasizing a regulatory role for metabolism in innate host defense ^107^. Metabolic inhibition was shown to reverse epigenetic changes in human monocytes in an *in vitro* model, characterized by decreased cytokine responses upon re-stimulation with microbial and metabolic stimuli ^104, 108^. Moreover, with parabiotic and chimeric mice intravenously vaccinated with BCG, BCG-educated hematopoetic stem cells were shown to generate epigenetically modified macrophages that provided enhanced protection against *M. tuberculosis* infection when compared with naïve macrophages ^102^. Such findings further point toward a potential therapeutic strategy that involves boosting such non-specific immunity to prevent *M. tuberculosis* infection. In a phase 2 randomized trial in South Africa, 990 QuantiferonTB Gold (QFT)-negative and HIV-negative adolescents who had undergone neonatal BCG vaccination received the H4:IC31 subunit vaccine, BCG revaccination, or placebo ^109^. The BCG vaccine reduced the rate of sustained QFT conversion with an efficacy of 45.4% ( *P* = 0.03) while the efficacy of the H4:IC31 vaccine was 30.5% ( *P* = 0.16) ^109^. Although it is unknown whether such findings hold true in other geographical settings, they do support a role for BCG revaccination and novel subunit candidate vaccines in inducing some level of non-specific protective immunity. Whether BCG revaccination can resuscitate the memory response in individuals to invoke similar protection observed in resisters needs to be systematically elucidated. Moreover, several distinct stable and transient changes are widely known to occur at the level of the epigenome that modulates the transcriptional landscape of immune cells. Unbiased approaches need to be employed to identify open chromatin regions and regulatory elements influencing histone modifications and differential DNA and RNA methylations in resisters, allowing the identification of important pathways that subsequently establish mycobacterial resistance.

## Outlook

The lack of an effective vaccine, poor diagnostics, and treatment management, along with the emergence of drug-resistant forms of *M. tuberculosis*, have sustained the global TB epidemic. Over the past 40 years, only two drugs of new classes have been approved by the US Food and Drug Administration, and pretomanid is the latest to be approved this year ^110^. Although several new drug candidates are in the clinical stages of development ^111^, the identification of pharmacological host targets that empower functional innate immune responses to rapidly restrict *M. tuberculosis* survival, without inducing cytotoxic effects, will provide novel adjuncts to antibiotic therapy. When classified using stringent criteria, infection resisters could represent about 5 to 15% of individuals among a given TB-endemic region and are invaluable targets for future research to decipher protective natural immunity. Inducing the right innate immune milieu that is likely present in resisters would enable the early and rapid elimination of *M. tuberculosis*, possibly instituting trained innate immune memory that can be investigated for vaccine strategies.

**Figure 1.**
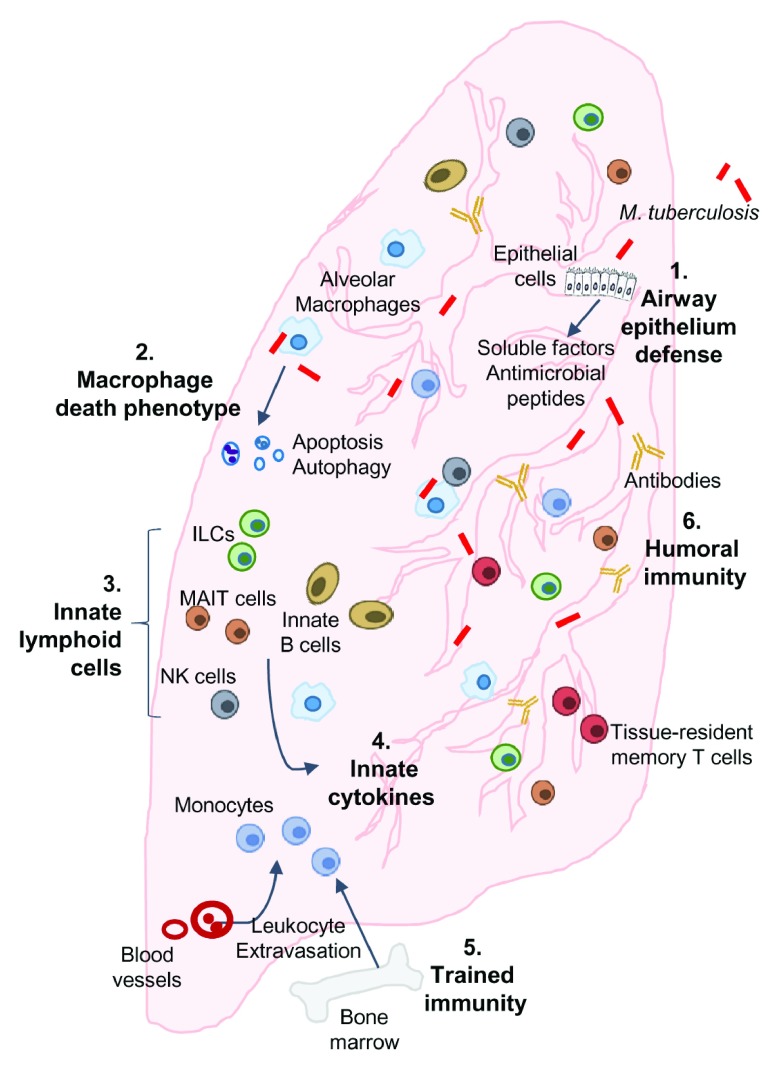
Overview of potential mechanisms and pathways contributing to the infection-resistant phenotype. Following exposure and infection of the airways and lung parenchyma with *Mycobacterium tuberculosis*, infection resisters may engage all or a combination of the following mechanisms and pathways to resist infection or rapidly clear infection: (1) airway epithelium defenses: secretion of soluble factors and anti-microbial peptides by airway epithelial cells; (2) macrophage-mediated *M. tuberculosis* growth restriction: programmed cell death or autophagy (or both) of lung-resident and recruited alveolar macrophages leading to intracellular restriction of *M. tuberculosis*; (3) innate lymphoid cells (ILCs): production of rapid and effective anti-mycobacterial responses by innate cell populations, including ILCs, mucosal-associated invariant T (MAIT) cells, natural killer (NK) cells, and innate B cells; (4) innate cytokine response: induction of cytokines that directly or indirectly control *M. tuberculosis* growth in macrophages; (5) trained immunity: molecular reprogramming of monocytes/macrophages leading to enhanced anti-mycobacterial responses; (6) humoral immunity: contribution of differentially glycosylated antibodies in restricting intracellular *M. tuberculosis*.

Recent observations from experimental analyses and mechanistic studies focused on various stages of initial infection to active TB disease offer crucial insights which are further complemented by large-scale TB-endemic cohort investigations that are aimed at interpreting the heterogeneity of host– *M. tuberculosis* interactions among resisters and infection-susceptible individuals. We have also discussed additional effector mechanisms that may be contributing to natural resistance, summarized in Figure 1. Emerging evidence further proposes that *M. tuberculosis* strains from various lineages evoke heterogeneic immune responses, associating with greater severity of disease and enhanced transmission ^112– 114^. Recently, our group reported that the transmissibility of strains belonging to the same lineage depends on their interaction with the host immune system leading to different trajectories in bacterial growth and in the development of disease pathology in the C3HeB/FeJ mouse model ^115^. This indicates that the role of immune variation in the host and the pathogen strain variation together may contribute to the infection-resistant and -susceptible phenotype in individuals exposed to *M. tuberculosis*. This is an area that awaits further investigation.
